# Subclinical Infection and Transmission of Clade 2.3.4.4 H5N6 Highly Pathogenic Avian Influenza Virus in Mandarin Duck (*Aix galericulata*) and Domestic Pigeon (*Columbia livia domestica*)

**DOI:** 10.3390/v13061069

**Published:** 2021-06-04

**Authors:** Sol Jeong, Jung-Hoon Kwon, Sun-Hak Lee, Yu-Jin Kim, Jei-Hyun Jeong, Jung-Eun Park, Weon-Hwa Jheong, Dong-Hun Lee, Chang-Seon Song

**Affiliations:** 1 Avian Disease Laboratory, College of Veterinary Medicine, Konkuk University, Seoul 05029, Korea; soljeong492@gmail.com (S.J.); junghoon.kwon@knu.ac.kr (J.-H.K.); cnescis@naver.com (S.-H.L.); yujinml@hanmail.net (Y.-J.K.); nar21ss@hanmail.net (J.-H.J.); 2 Laboratory of Veterinary Microbiology, College of Veterinary Medicine, Kyungpook National University, Daegu 41566, Korea; 3 Wildlife Disease Response Team, National Institute of Wildlife Disease Control and Prevention, Gwangju 62407, Korea; jepark87.11@gmail.com (J.-E.P.); purify@korea.kr (W.-H.J.); 4 Department of Pathobiology and Veterinary Science, The University of Connecticut, Storrs, CT 06269, USA

**Keywords:** highly pathogenic avian influenza, H5N6, clade 2.3.4.4, pathogenicity, wild bird, mandarin duck, pigeon

## Abstract

Since 2014, H5Nx clade 2.3.4.4 highly pathogenic avian influenza viruses (HPAIV) have caused outbreaks in wild birds and poultry in multiple continents, including Asia, Europe, Africa, and North America. Wild birds were suspected to be the sources of the local and global spreads of HPAIV. This study evaluated the infectivity, pathogenicity, and transmissibility of clade 2.3.4.4 H5N6 HPAIV in mandarin ducks *(Aix* *galericulata*) and domestic pigeons (*Columbia livia domestica*). None of the birds used in this study, 20 mandarin ducks or 8 pigeons, showed clinical signs or mortality due to H5N6 HPAI infection. Two genotypes of H5N6 HPAIV showed replication and transmission by direct and indirect contact between mandarin ducks. H5N6 HPAIV replicated and transmitted by direct contact between pigeons, although the viral shedding titer and duration were relatively lower and shorter than those in mandarin ducks. Influenza virus antigen was detected in various internal organs of infected mandarin ducks and pigeons, indicating systemic infection. Therefore, our results indicate mandarin ducks and pigeons can be subclinically infected with clade 2.3.4.4 H5N6 HPAIV and transfer the virus to adjacent birds. The role of mandarin ducks and pigeons in the spread and prevalence of clade 2.3.4.4 H5N6 viruses should be carefully monitored.

## 1. Introduction

After the discovery of A/goose/Guangdong/1/1996(H5N1) (Gs/GD), a highly pathogenic avian influenza (HPAI) virus in China, global spread of Gs/GD-lineage H5 HPAI viruses has been observed, and the viruses have evolved into phylogenetically distinct clades (0–9) and subclades by genetic drift and shift [1]. Before 2008, H5N1 was the major subtype of the Gs/GD lineage; however, novel H5Nx subtypes, such as H5N2, H5N5, H5N6, and H5N8, have emerged as a result of reassortment with prevailing low-pathogenic avian influenza viruses (LPAI) [2,3,4]. Since 2014, H5N6 and H5N8 subtypes of viruses bearing the hemagglutinin (HA) gene of clade 2.3.4.4 have been detected in wild birds and poultry in multiple continents, including Asia, Europe, Africa, and North America. They have become the dominant subtypes of the Gs/Gd lineage [5,6,7,8,9,10].

After the initial emergence from China in 2013, clade 2.3.4.4 H5N6 HPAI virus spread to Laos and Vietnam during 2014–2015, eventually being well established in the countries [10,11,12], and more eastward to Japan and South Korea during 2016–2017 [13,14]. The clade 2.3.4.4 HPAI H5N6 viruses, which have been the dominant subtype in China, have evolved into multiple genotypes and caused infection in multiple species, including mammalian species and humans [15,16,17,18]. From 2014 to March 2021, 30 cases of laboratory-confirmed human infection by H5N6 HPAIV, including 8 fatal cases, have been reported to the World Health Organization (WHO) [19]. There is no evidence for human-to-human transmission of clade 2.3.4.4 H5N6 HPAI virus, but the infected poultry was suspected as a source of human infection. Therefore, the spread of clade 2.3.4.4 H5N6 viruses could be a severe threat to the poultry industry and public health.

In the winter of 2016, the novel reassortant clade 2.3.4.4 H5N6 HPAIV was isolated from the feces of wild mandarin ducks in South Korea [20], and its spread to poultry farms resulted in the depopulation of more than 25 million poultry in Korea from October 2016 to May 2017. The clade 2.3.4.4 H5N6 HPAIV that caused outbreaks during 2016–2017 contained the neuraminidase (NA) gene derived from the H6N6 LPAI virus and the internal genes from LPAI viruses, including H9N2 and H7N9 subtypes [15,21]. Subsequently, six distinct genotypes, C1–C6, of H5N6 HPAIV that bear different polymerase acidic (PA) and nonstructural (NS) genes and are highly similar to the LPAI viruses from Eurasia were reported in Korea and Japan during 2016–2017 [13,14]. Genotype C1 was first detected in wild birds in Korea; however, genotype C4 became predominant in wild birds and poultry during epidemics in Korea [14]. The clade 2.3.4.4 H5N6 HPAI viruses have caused outbreaks in poultry in China as a major subtype also causing human infection [22], but this strain has not been identified in Korea since 2017.

This study evaluated the infectivity, pathogenicity, and transmissibility of genotypes C1 and C4 of clade 2.3.4.4 H5N6 HPAI in mandarin ducks, a representative reservoir of clade 2.3.4.4 H5Nx HPAIV, to understand the role of the species in the maintenance and circulation of the virus. Mandarin ducks are both migrants and terrestrial residents in East Asia; some inhabit East Asia, including eastern Russia, China, the Korean Peninsula, and Japan, throughout the year. Some are passage migrants that move within East Asia and Russia [23]. The identification of HPAI H5Nx viruses in mandarin ducks has been recurrently reported in Korea, Japan, and China from 2010 to 2020 [20,24,25,26,27,28,29,30]. In particular, the clade 2.3.4.4 HPAI H5N6 virus in Korea in 2016 was first identified in mandarin ducks [20].

Furthermore, we also evaluated the infectivity, pathogenicity, and transmissibility of genotype C1 virus in domestic pigeons, which are common terrestrial bird species that play an intermediate role in transmitting HPAIV between wild birds and poultry [31]. Pigeons have not been considered an important intermediate host species of HPAI as they have been reported to be resistant to HPAI infection in previous studies [32,33,34,35]. However, the susceptibility to some H5 HPAI viruses observed in recent experimental studies and natural infection cases [36,37,38,39,40,41,42] indicates the potential changes in virulence of recent HPAI viruses in pigeons.

## 2. Materials and Methods

### 2.1. Animals

Twenty-four mandarin ducks (*Aix galericulata*) and twelve domestic pigeons (*Columbia livia domestica*) were purchased from a live bird market. The birds that tested negative for the presence of antigens of and antibodies against the influenza virus were used for the experiment. Oral and cloacal swabs were screened for influenza virus by real-time reverse transcription-polymerase chain reaction (rRT-PCR) [43]. Serum samples were analyzed for influenza type A nucleoprotein (NP)-specific antibody using a commercial competitive NP-ELISA kit (Bionote, Hwaseong, Korea) according to the manufacturer’s instructions and for HA-specific antibody using hemagglutination inhibition (HI) assay according to the guidelines of the World Organization for Animal Health (OIE) [44].

All birds were kept in self-contained isolation units ventilated under negative pressure with HEPA-filtered air. Commercial chicken feed and freshwater were provided daily. All animal experiments were conducted in a biosafety level 3 (BSL-3) facility at Konkuk University.

### 2.2. Viruses

Two genotypes, C1 (A/mandarin duck/Korea/K16-187-3/2016(H5N6), GISAID EpiFlu Isolate ID EPI_ISL_238148) and C4 (A/duck/Korea/ES2/2016(H5N6), GISAID EpiFlu Isolate ID EPI_ISL_239262), which belong to clade 2.3.4.4, were used. The viruses passaged twice in embryonated chicken eggs were used for the animal experiments. Virus titration was conducted using 9- to 11-day-old embryonated chicken eggs by the Reed–Muench method [45] to determine the 50% egg infectious dose (EID_50_)/mL.

### 2.3. Experimental Design

Both genotypes C1 and C4 were used to infect mandarin ducks. Twenty-four mandarin ducks were divided into two groups, and each group was divided into three subgroups: seven for inoculation, three for contact exposure, and two for indirect exposure. Genotype C1 was used to infect pigeons experimentally. Ten pigeons were divided into two groups: seven for inoculation and three for contact exposure.

Fourteen mandarin ducks and seven pigeons in the inoculation groups were inoculated with 0.2 mL of 10^7.0^ EID_50_/mL via choanal cleft. To evaluate transmissibility through contact, three naïve birds from each group were co-housed with the inoculated birds 24 h later. We used a cage with two rooms divided by a barrier with a window of 3-layered wire mesh (30 × 35 cm) to evaluate the transmissibility through indirect transmission, allowing common and indirect airflow. The two outer layers had 1.2 cm and an inner layer had 2 mm of grid size which can prevent direct contact between the two rooms. Two inoculated and naïve mandarin ducks were housed in each room of a mesh-divided cage. All birds were monitored daily for clinical signs and mortality.

### 2.4. Viral Quantification

To quantify viral shedding, oropharyngeal and cloacal swabs were collected from the birds at 2, 4, 6, 8, and 12 days post-inoculation (dpi) and suspended in 1 mL of phosphate-buffered saline (PBS) containing antibiotics (400 mg/mL of gentamicin). Viral RNA was extracted from 200 µL of the suspension using MagNA Pure 96 DNA and Viral NA Small Volume Kit on the MagNA Pure 96 instrument (Roche Applied Sciences, Penzberg, Germany) according to the manufacturer’s instructions. Quantitative analysis of viral RNA was conducted using the cycle threshold (Ct) value using rRT-PCR targeting the M gene [43]. It has been demonstrated that Ct values correlate strongly with infectious viral titer [46]. To convert the Ct value into the EID_50_ equivalent unit, we generated a standard curve between them as previously described [36]. Briefly, the HPAI H5N6 challenge virus with a known EID_50_ titer was serially diluted 10-fold and quantified using rRT-PCR as described above. Ct values of viral 10-fold dilutions were plotted against viral EID_50_ titers. The resulting standard curve showed a high correlation (r^2^ > 0.99). The detection limit was 10^2^ EID_50_ equivalent/mL. Virus titers in samples were calculated by extrapolation of the standard curve equation.

### 2.5. Statistical Analysis

The presence of statistically significant differences in the viral shedding titers was identified using one-way ANOVA with Tukey’s post hoc test in GraphPad Prism 5 software (GraphPad Software Inc., San Diego, CA, USA). The samples from which viral shedding was not detected by rRT-PCR were treated as 10^1.9^ EID_50_ equivalent/mL for statistical purposes. The *p*-values of < 0.05 were regarded as statistically significant.

### 2.6. Serology

To verify seroconversion, serum samples were collected from the birds at 1 day before and 14 days after the infection. For homologous anti-H5 antibody detection, the serum samples were treated with receptor-destroying enzyme (Denka Seiken Co, Ltd., Gosen, Japan) according to the manufacturer’s instructions and screened for HI antibody. Formalin-inactivated homologous antigens and 1% chicken red blood cells were used in the HI assay. A commercially available competitive NP-ELISA kit (Bionote) was used to detect anti-influenza A NP-specific antibodies according to the manufacturer’s instructions.

### 2.7. Immunohistochemistry

At 5 dpi, two birds from each inoculation group were euthanized. Organs (brain, trachea, heart, lung, liver, spleen, pancreas, kidney, cecal tonsil, and intestine) were sampled, fixed in 4% paraformaldehyde, and embedded in paraffin. The resulting blocks were cut into 5 µm-thick sections and subjected to immunohistochemical staining to detect influenza virus-specific signals, as described previously [47]. The tissue sections were incubated overnight at 4 ℃ with goat anti-influenza A virus antibody (25 µg/mL, Merck, Darmstadt, Germany), for 2 h at room temperature with biotinylated anti-goat IgG (Vector Laboratories Inc., Burlingame, CA, USA), and for 1 h at room temperature with horseradish peroxidase-conjugated streptavidin (Vector Laboratories Inc.). Positive signals were visualized using diaminobenzidine and counterstained with methyl green. Negative control staining was performed by omitting the primary antibody incubation step.

## 3. Results

### 3.1. Clinical Signs of Infection, Viral Shedding, and Transmission

No mortality or clinical signs were observed in both mandarin ducks and pigeons (Table 1), except for a pigeon in the inoculation group that died at 13 dpi. The pigeon did not exhibit any clinical signs of HPAI, gross lesion at autopsy, or viral shedding. Thus, it was difficult to conclude whether HPAI-related fatality was present.

**Figure 1:**
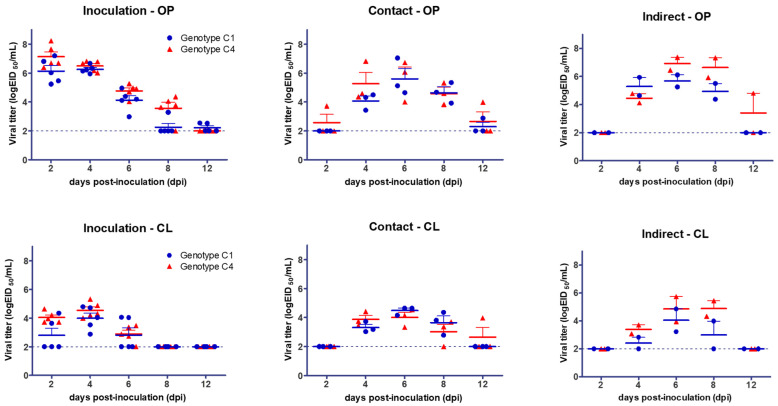
Viral shedding of mandarin ducks infected with genotypes C1 and C4 of clade 2.3.4.4 H5N6 HPAIV. The detection limit was 10^2^ EID_50_ equivalent/mL (dotted line). OP, oropharynx; CL, cloaca; blue dot, genotype C1; red triangle, genotype C4.

Both genotypes C1 and C4 were able to efficiently replicate in all mandarin ducks used in the experiment. Successional peak viral titers were in the order of inoculation, contact exposure, and indirect exposure, indicating that both genotype C1 and C4 clade 2.3.4.4 H5N6 HPAI viruses can successfully replicate in mandarin ducks and be transmitted through direct contact as well as indirect exposure (Table 1, Figure 1). However, excretions of the genotype C1 virus were detected from 2 to 12 dpi in inoculated birds, and the genotype C4 virus was detected from 2 to 8 dpi in inoculated birds. The peak amount of viral shedding was 10^7.19^ EID_50_/mL and 10^8.23^ EID_50_/mL for genotypes C1 and C4, respectively. Both contact- and indirectly exposed birds excreted detectable amounts of viruses from 4 dpi, except one bird in the contact exposure group for the C4 genotype, which excreted the virus via the oropharynx from 2 dpi. These results show that both genotypes C1 and C4 can replicate in and transmit between mandarin ducks via direct or indirect exposure. The viral shedding titers were not significantly different between the viral genotypes or sample types possibly due to the limited number of animals used in the study.

**Figure 2:**
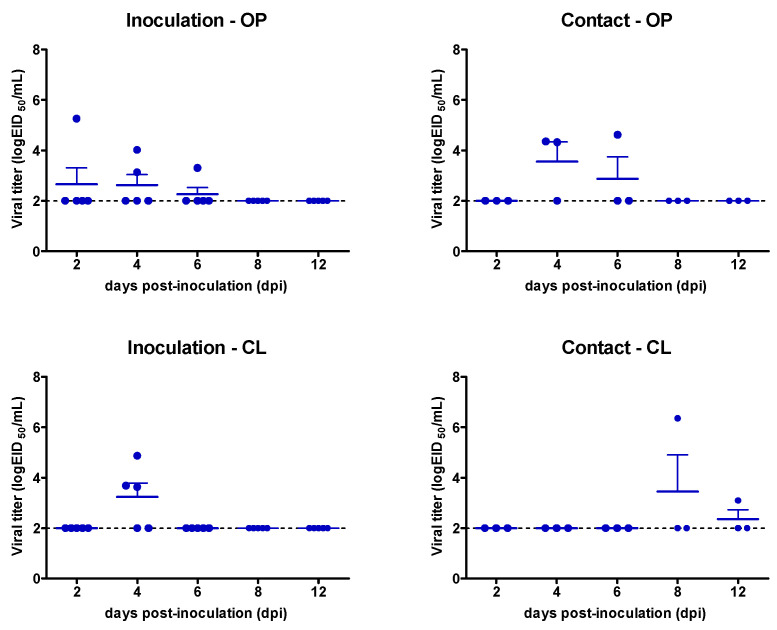
Viral shedding of domestic pigeons infected with genotype C1 of clade 2.3.4.4 H5N6 HPAIV. The detection limit was 10^2^ EID_50_ equivalent/mL (dotted line). OP, oropharynx; CL, cloaca.

Domestic pigeons exhibited lower levels of or no viral shedding compared to mandarin ducks. Three out of five birds (60%) in the inoculation group and two out of three (66.67%) in the contract exposure group of domestic pigeons showed viral shedding (Table 1). Viral shedding was detected from 2 to 6 dpi in the inoculation group (Figure 2). The peak amount of viral shedding was 10^5.26^ EID_50_/mL and 10^6.35^ EID_50_/mL for the inoculation and contact exposure groups, respectively. One contact-exposed pigeon shed the virus from 4 to 12 dpi, with a peak viral titer of 10^6.4^ EID_50_/mL at 8 dpi. The other pigeon in the contact group shed the virus only at 4 dpi via the oropharynx. These results show that the clade 2.3.4.4 H5N6 HPAI virus can replicate in domestic pigeons and transmit through direct contact.

### 3.2. Serology

Seroconversion was confirmed in all mandarin ducks by NP-ELISA and HI assay. Seroconversion was not detected by NP-ELISA in one mandarin duck in the inoculation group of genotype C4; however, the results of viral detection by rRT-PCR and the HI assay indicate viral replication in the bird (Table 1).

In the HI assay, only one pigeon in the inoculation group showed seroconversion. However, seroconversion in all pigeons in the inoculation group and one out of three in the contact exposure group was confirmed by NP-ELISA, indicating viral replication and transmission via direct contact in pigeons (Table 1).

### 3.3. Immunohistochemistry

**Figure 3:**
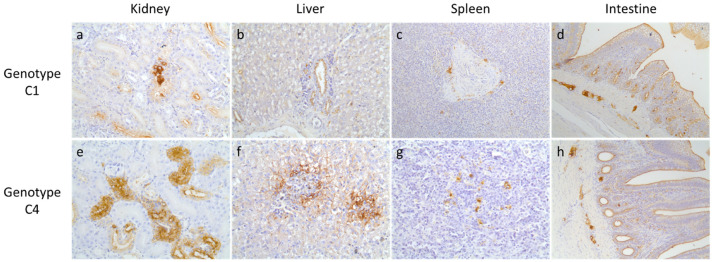
Immunohistochemistry of organs from mandarin ducks. Influenza-specific signals were detected by diaminobenzidine (brown precipitate) in the (**a**) kidney (400×), (**b**) liver (400×), (**c**) spleen (200×), and (**d**) intestine (200×) of mandarin ducks infected with genotype C1 of clade 2.3.4.4 H5N6 HPAIV, and the (**e**) kidney (400×), (**f**) liver (400×), (**g**) spleen (400×), and (**h**) intestine (200×) of mandarin ducks infected with genotype C4 of clade 2.3.4.4 H5N6 HPAIV.

**Figure 4:**
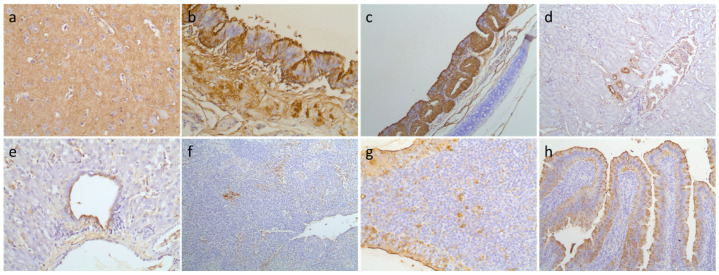
Immunohistochemistry of organs from domestic pigeons. Influenza-specific signals were detected by diaminobenzidine (brown precipitate) in the (**a**) brain (400×), (**b**) lung (400×), (**c**) trachea (200×), (**d**) kidney (200×), (**e**) liver (400×), (**f**) spleen (200×), (**g**) cecal tonsil (400×), and (**h**) intestine (200×) of domestic pigeons infected with H5N6 genotype C1.

In the immunohistochemical (IHC) analysis, positive signals for influenza virus from various organs indicated systemic tissue tropism of the clade 2.3.4.4 H5N6 HPAIV in mandarin ducks and domestic pigeons. Influenza virus was localized in all tested organs except for the lungs of genotype C1-infected mandarin ducks, and the heart of genotype C4-infected mandarin ducks and genotype C1-infected domestic pigeons (Table 2, Figure 3 and Figure 4). Influenza antigen was not detected in any of the negative control tissue sections. Due to the limited number of birds used for this study, it is not possible to confirm that the viruses have no tropism in the organs negative for an influenza signal in the IHC analysis.

## 4. Discussion

Based on the viral genome sequence data in the GISAID database (https://platform.gisaid.org/, accessed on 19 April 2021), clade 2.3.4.4 H5N6 HPAI viruses have been detected in multiple species of wild birds, including mandarin duck (*Aix galericulata*), northern pintail (*Anas acuta*), Eurasian wigeon (*Anas penelope*), common teal (*Anas crecca*), heron (*Ardeidae sp.*), and domestic pigeon (*Columbia livia domestica*) in many other countries in Asia, including Japan, China, and Vietnam. Clade 2.3.4.4 H5Nx HPAI viruses have been detected in multiple continents since 2014. Wild birds have been suggested to play a major role in the prevalence, evolution, and dissemination of the viruses. Seasonal movement between and aggregation in the breeding and wintering sites of migratory wild birds have contributed to the long-distance dissemination and reassortment of HPAI viruses [48,49,50]. Subclinical infection of wild birds with low levels of clinical signs and prolonged viral excretion may facilitate efficient conveyance of the virus to unaffected areas or other susceptible hosts. Terrestrial birds can freely interact with wild birds and poultry through water and food sources and serve as potential hosts and bridge species for HPAI transmission. Detections of clade 2.3.4.4 H5N6 HPAI virus in wild migratory or terrestrial birds and subclinical infections in these birds are of concern due to the possibility that these birds could maintain the virus and further spread outside of Asia.

This study investigated the pathogenicity and transmissibility of clade 2.3.4.4 H5N6 HPAI in mandarin ducks and domestic pigeons. In mandarin ducks, both genotypes C1 and C4 of clade 2.3.4.4 H5N6 HPAIV successfully replicated and induced seroconversion. Transmission to naïve birds via direct and indirect exposure was also observed. None of the mandarin ducks exhibited clinical signs or mortality. Similar to this study, mandarin ducks experimentally infected with clade 2.3.4.4 H5 HPAIV in other studies exhibited viral shedding without clinical signs and transmitted the virus to naïve birds [36,51,52]. The results indicate that the mandarin duck could serve as a healthy carrier of clade 2.3.4.4 H5N6 viruses. Considering their geographical distribution, the role of mandarin ducks in the spread of HPAIV would be limited to East Asia; however, they can be a source of infection for other wild waterfowl, serving long-distance transmission of HPAIV.

Genotype C1 of clade 2.3.4.4 H5N6 HPAIV also infected domestic pigeons without clinical signs or mortality. Viral transmission by direct contact was observed in two pigeons. In one of them, the viral shedding lasted until 12 dpi. Although the viral propagation and shedding period were higher and prolonged in mandarin ducks, domestic pigeons exhibited substantial viral shedding, thereby transmitting the infection to naïve birds, indicating that pigeons may be potential carriers of clade 2.3.4.4 H5N6 HPAI virus. Seroconversion and distribution of the viral antigen after infection also indicate that pigeons can be systemically infected by clade 2.3.4.4 H5N6 HPAI virus.

Seroconversion was confirmed in all inoculated and one contact-exposed pigeon by NP-ELISA; however, H5-specific antibody was detected in one pigeon by HI assay. Previous studies reported similar results that the HI test had lower sensitivity than the commercially available competitive NP-ELISA for non-gallinaceous species [33,53], suggesting an underestimation of the immune response in AIV-exposed pigeons.

Influenza virus antigens were detected in many internal organs of mandarin ducks and pigeons infected with clade 2.3.4.4 H5N6 HPAI viruses, indicating systemic infection. Compared to previous experimental studies in mandarin ducks and pigeons with clade 2.3.4.4 H5N8 HPAI virus [36], clade 2.3.4.4 H5N6 HPAI virus antigen was detected in various organs tested, including the brain. Clade 2.3.4.4 H5N6 HPAI exhibited neurotropism, consistent with previous studies [35,37,39,54]. Despite the systemic distribution of the virus in mandarin ducks and pigeons, the birds did not show any detectable clinical signs in this study.

Pigeons are potential intermediate hosts of H5 HPAIV with less possibility of effective viral propagation and transmission than species of *Anseriformes* or *Galliformes* and are suggested not to play a significant role in the maintenance of the virus [32,33,34,35]. Clade-dependent differences in pathogenicity and mortality in the course of H5 HPAI infection have been reported in previous experimental infection studies [36,37,38,39,40,41,55], and natural infection cases of H5 HPAIV in pigeons have been identified [56,57,58]. However, few studies have reported intra-species transmission of the H5 HPAIV in pigeons after infection [42]. In our previous experiment using clade 2.3.4.4 H5N8 HPAI, viral shedding was detected in infected pigeons; however, transmission was not detected [36]. In contrast to previous studies on the infectivity of HPAI H5 viruses in pigeons, experimental infection of clade 2.3.4.4 HPAI H5N6 viruses in pigeons in this study resulted in replication and transmission by direct contact. However, the role of feral pigeons in AIV ecology needs to be further elucidated owing to limited susceptibility and transmissibility. Further development and subsequent alteration in the biological characteristics of clade 2.3.4.4 HPAI H5Nx viruses need to be investigated closely, particularly for clade 2.3.4.4 HPAI H5N6 viruses, which have caused multiple outbreaks in birds and humans.

The pathogenicity and transmissibility of HPAIV can be influenced by both host and viral factors and significantly affect viral ecology. Variable susceptibility to HPAI infection depending on host species has been reported [37,59,60,61]. In contrast to Gs/GD H5N1 HPAIV, which can efficiently replicate and induce disease with various symptoms in wild birds and domestic poultry [62], clade 2.3.4.4 H5Nx HPAIV exhibited mild or no clinical signs of infection in wild waterfowl [63,64]. This suggests better adaptation of clade 2.3.4.4 H5Nx HPAIV to waterfowl and a consequent influence on long-distance dispersal by wild migratory birds. The potential adaptation to a specific host species may lead to decreased pathogenicity, increased viral shedding, changes in the viral shedding pattern or duration, or a decreased infectious dose [63]. Clade 2.3.4.4 H5N6 HPAIV with different internal gene origins may exhibit altered pathogenicity in avian and mammalian animal models [65]. Therefore, the emergence of novel genotypes due to reassortment should be carefully monitored during HPAIV outbreaks, and possible alterations in pathogenicity and transmissibility in susceptible host species need to be evaluated. Subclinical infection and replication of clade 2.3.4.4 H5N6 HPAIV in mandarin ducks and domestic pigeons, and reports of human infection with highly similar H5N6 viruses, including fatal cases from China, highlight the potential for the maintenance and dissemination of zoonotic H5N6 viruses to distant geographical areas. Previous HPAI surveillance strategies in Korea focused on migratory waterfowl. However, HPAI infections in wild terrestrial birds are of concern as they might facilitate the transmission of viruses between wild and domestic birds. Considering the possibility of potential dispersal and maintenance of HPAI viruses through these wild bird species, enhanced active surveillance in both migratory and terrestrial wild birds should be implemented.

## Figures and Tables

**Table 1:** Summary of clinical signs, viral shedding, and serology in mandarin ducks and domestic pigeons infected with clade 2.3.4.4 H5N6 HPAIV.

Species	Viral Genotype	Group	Clinical Sign (Sick/Total)	Mortality (Dead/Total)	Viral Shedding (Max Mean Viral Titer ^1^ and dpi)	Serology	
						NP-ELISA (Mean PI ^2^)	HI Assay (Mean Titer)
Mandarin duck	C1	Inoculation	0/5	0/5	5/5 (6.26, 4 dpi)	5/5 (93.91)	5/5 (5.2)
		Contact	0/3	0/3	3/3 (5.60, 6 dpi)	3/3 (93.42)	3/3 (5.67)
		Indirect	0/2	0/2	2/2 (5.69, 6 dpi)	2/2 (92.16)	2/2 (5.5)
	C4	Inoculation	0/5	0/5	5/5 (7.13, 2 dpi)	4/5 (95.56)	5/5 (5.2)
		Contact	0/3	0/3	3/3 (5.60, 6 dpi)	3/3 (95.47)	3/3 (4.33)
		Indirect	0/2	0/2	2/2 (6.91, 6 dpi)	2/2 (94.37)	2/2 (6)
Domestic pigeon	C1	Inoculation	0/5	1/5	3/5 (3.24, 4 dpi)	4/4 ^3^ (93.65)	1/4 ^3^ (6)
		Contact	0/3	0/3	2/3 (3.56, 4 dpi)	1/3 (67.05)	0/3

^1^ logEID_50_/mL. ^2^ Percent inhibition. ^3^ One pigeon died at 13 dpi before bleeding for serological analysis.

**Table 2:** Immunohistochemistry for influenza virus detection in mandarin ducks and domestic pigeons.

Species	Viral Genotype		Brain	Heart	Trachea	Lung	Liver	Pancreas	Intestine	Kidney	Spleen	Cecal Tonsil
Mandarin duck	C1	Bird 1	+	−	+	−	+	−	+	+	+	+
		Bird 2	+	+	+	−	+	+	+	+	+	+
	C4	Bird 1	+	−	+	+	+	+	+	+	+	+
		Bird 2	−	−	+	+	+	+	+	+	+	+
Domestic pigeon	C1	Bird 1	+	−	+	+	+	+	+	+	+	+
		Bird 2	+	−	+	+	+	−	+	+	+	+
